# Determination of aflatoxin levels in bokina beverage

**DOI:** 10.4314/gmj.v55i4.10

**Published:** 2021-12

**Authors:** Ebenezer Ofori-Attah, Abigail Aning, Mark Ofosuhene, Justice Kumi, Regina Appiah-Opong

**Affiliations:** Department of Clinical Pathology, Noguchi Memorial Institute for Medical Research, College of Health Sciences, University of Ghana, Legon, Ghana

**Keywords:** Aflatoxin, Bokina, Contamination, Milk, Millet

## Abstract

**Objective:**

The main aim of this study was to investigate levels of total aflatoxin and aflatoxin M_1_ in bokina, a home-made non-alcoholic beverage prepared from dairy milk, millet and sugar.

**Methods:**

Bokina, dairy milk and millet were purchased monthly over a period of 7 months from bokina producers at Ashaiman and Nima, in Ghana. Total aflatoxin and aflatoxin M_1_ levels in these samples were measured using a fluorometric procedure and High-Performance Liquid Chromatography.

**Results:**

Aflatoxin levels in bokina samples ranged from 1.0 to 21.0 ppb for Ashaiman samples and 1.0 to 23.0 ppb for Nima samples. Out of 21 samples from each site 1 from Ashiaman and 2 from Nima had levels total aflatoxin above the acceptable limit of 20 ppb. Similarly, total aflatoxin levels millet samples ranged from 1.0 to 55.0 ppb for Ashaiman and 5.0 to 53.0 ppb for Nima samples, with 2 samples from Ashiaman and 6 from Nima having levels above 20ppb. The levels of Aflatoxin M_1_ in milk ranged from 0.09 to 6.20 ppb for Ashaiman samples and 0.13 to 12.55 ppb for Nima samples. Out of the samples, 12 from Ashiaman and 10 from Nima (n=21) had levels of Aflatoxin M_1_ above the acceptable limit of 0.5 ppb.

**Conclusion:**

Bokina samples tested were contaminated with aflatoxin. All doses of aflatoxin have a cumulative effect on the risk of cancer. Therefore, farmers and bokina producers must be educated on good storage practices and monitored to protect the public from aflatoxin exposure and toxicity.

**Funding:**

The study was self-funded

## Introduction

Aflatoxins (B_1_, B_2_, G_1_ and G_2_) are a group of highly toxic metabolites produced by the fungi *Aspergillus flavus* and *Aspergillus parasiticus*.^1^ Aflatoxin B_1_ (AFB_1_) is the most toxic and potent carcinogen that occurs naturally in various foods such as maize, groundnuts, millet, rice and other grains.^2^,^3^

Once ingested, aflatoxin B1 is metabolized primarily by mixed-function oxidases of the liver to aflatoxin 8, 9-epoxide and various metabolites including Aflatoxin M_1_ (AFM_1_).^4^ This epoxide is electrophilic and binds covalently to hepatocytes. Consumption of foodstuffs contaminated by aflatoxins may lead to hepatotoxic or carcinogenic effects. Acute aflatoxin exposure is toxic to the liver and may cause death in a few days.^5^,^6^ Chronic exposure to aflatoxin could also result in liver cancer in individuals infected with the hepatitis B virus.^4^ On the other hand, this exposure is reported to compromise immunity and interfere with the metabolism of proteins and multiple micronutrients that are critical to health and growth in children.^7^

According to the United Nations Food and Agriculture Organization^8^ 25% of world food crops are contaminated with aflatoxins, and countries that are situated between 40oN and 40oS are most at risk, potentially up to 5 billion people in the developing world including Ghana. This estimated level of aflatoxin contamination significantly underestimates occurrence above the detectable levels (up to 60–80%).^8^ Under optimum temperature conditions (24–27°C) and humidity >12%, *Aspergillus flavus* and *Aspergillus parasiticus* can grow and produce aflatoxin on nearly all agricultural commodities.^9^,^10^ The internationally acceptable level of aflatoxin in human foods is less than twenty parts per billion (20 ppb).^11^,^12^,^13^,^14^ Aflatoxin M_1_, a hydroxylated metabolite of AFB_1_ and can be detected in dairy products from animals that have ingested food contaminated with Aflatoxin B_1_.^15^

Although the metabolite AFM_1_ from AFB_1_ is less toxic, acceptable levels of AFM_1_ in milk for human consumption is 0.5 ppb.

This is possibly because milk is consumed primarily by children during the early developmental stages when the immune system is more susceptible to the suppressive effects^4^ Similar to other aflatoxins, AFM_1_ can survive pasteurization, thus human exposure to this toxin through dairy products such as milk is particularly of great concern since infants feed on it constantly.^16^,^17^,^18^

Bokina is a homemade non-alcoholic beverage prepared from millet and dairy milk. Millet is a very good source of dietary fibre, micronutrients and polyphenols.^19^ Millet could be used to develop nutritious weaning food products to benefit several communities. It could also be processed into instant, low-cost, nutritious food suitable for households and commercial purposes.^20^,^21^ Dairy milk is also important as a rich source of protein for the good health of children and adults. Dairy milk has been chosen as a carrier for supplementation of Vitamins A and D where these are generally deficient in the diet of children.^22^,^23^

Bokina is quite popular in Ghana and consumed by both adults and children. It could be used as weaning food for children of ages above one year due to its nutritional value. However, the safety of this beverage on the market, with regards to aflatoxin levels had not yet been reported. The local production and sale of bokina in communities including Nima and Ashaiman is currently one of the fast-growing businesses in the region. In this study, we investigated the aflatoxin content of homemade bokina in selected communities in the Greater Accra region of Ghana.

## Methods

### Study site

The study sites were in Ashaiman metropolitan area and Nima in the Accra metropolitan area where the beverage is produced in large quantities before distribution for sale.

### Materials

VICAM AflaTest equipment (Source Scientific, CA, USA) with a fluorescent detector was used to determine total aflatoxin (B_1_, B_2_, G_1_ and G_2_) levels according to the method described by the Association of Official Analytical Chemists' (AOAC official method 993.31, V1 series 4, 1999). AflaTest immunoaffinity columns were purchased from VICAM, (MA, USA). All chemicals used were of analytical grade and obtained from standard suppliers.

### Ethical approval

Ethical approval was not required for the study because there was no direct participation of human subjects or animals.

### Sample collection

Over a period of 7 months, 500 ml bokina, 500 ml milk and 100 g millet were purchased monthly from bokina producers at three different locations each in Ashaiman and Nima. The samples were kept in clean containers, labeled, and transported to the Department of Clinical Pathology, Noguchi Memorial Institute for Medical Research and stored at -20°C until the analysis was performed.

### Sample processing and analysis

Levels of total aflatoxin were determined as described by AOAC (1999). Aflatoxin extraction was performed by adding 5 g NaCl and 100 ml of 80% methanol to 50 g of millet, 50 ml of bokina and 50 ml of milk separately in a blender and blend for 2 min at high speed (1500 rpm). Subsequently, each extract was filtered twice; first through fluted filter paper and then with a glass microfiber filter (90 mm, 1 µm). The eluents were collected into beakers. Distilled water (40 ml) was added to 10 ml of each of the eluents and mixed thoroughly. The resulting mixture was filtered through a glass microfiber filter. Ten milliliters aliquots of the filtered mixtures were passed through Aflatest columns separately, at a rate of 1–2 drops/second. The columns were washed twice with 10 ml distilled water at the rate of 1–2 drops/second. This was followed by elution into glass cuvettes with 1 ml methanol at the rate of 1–2 drops/second.

### Determination of total aflatoxin content

Measurement of the total aflatoxin concentration was performed after 1 ml of Aflatest developer was added to the filtered mixtures and mixed thoroughly. Detection was done with the fluorometer (VICAM, series 4, detection limit 0.50 ppb) at the wavelength of excitation 363 nm and emission 440 nm.

### Aflatoxin M_1_ content analysis by high-performance liquid chromatography

Aflatoxin M_1_ levels in bokina and milk were analyzed using high-performance liquid chromatography (HPLC) techniques as described with slight modification. Samples were analyzed using reverse-phase HPLC (Shimadzu Prominence model; Kyoto, Japan) consisting of a binary solvent delivery system (LC-20AB), a degasser (DGU-20A3), an auto-sampler (SIL-20ACHT), a column temperature controller (CTO - 10AS VP) and a fluorescence detector (RF-10AXL).^5^ The latter was set at a wavelength of excitation 360 nm and emission 440 nm. The mobile phase consisted of 45% methanol and 55% water and the flow rate was 1 ml/min. A C18 column (Tskgel ODS, diameter 5 µm, length x width, 150 mm ×4.6 mm) was used. The column temperature was maintained at 40°C and the injection volume was 20 µl.

## Results

Aflatoxins were detected in all the samples as shown in Table 1. Few samples from Ashaiman and Nima had total aflatoxin (AF) levels above the acceptable limit (>20 ppb). Six (28.6%) of the millet samples from Nima, had total aflatoxin levels that were above the acceptable limit. Most of the milk samples collected had AFM_1_ levels above the acceptable limit of 0.5 ppb (Table 1).

**Table 1:** Total Aflatoxin levels in millet and bokina and Aflatoxin M_1_ levels in milk

*Sample*	Ashaiman (n=21)	Range (ppb)	Nima (n=21)	Range (ppb)
*Bokina (AF)*	1	1.0–21.0	2	1.0–23.0
*Millet (AF)*	2	1.0–55.0	6	5.0–53.0
*Milk (AFM_1_)*	12	0.09–6.20	10	0.13–12.55

Columns 2 and 4 show the number of samples with aflatoxin levels above the acceptable limit. Ranges of aflatoxin levels recorded in the samples are shown in columns 3 and 5.

**Figure 1:**
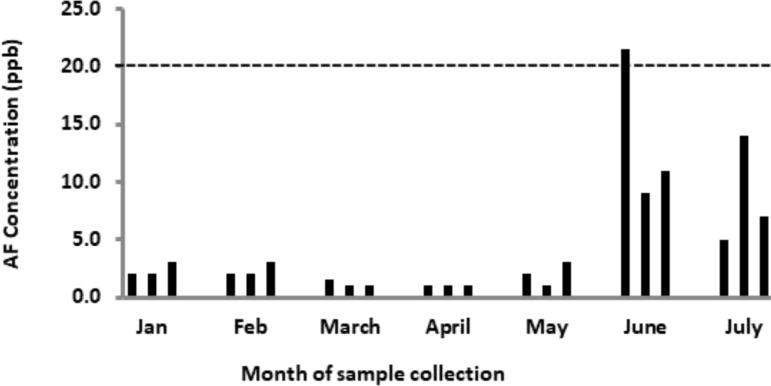
Aflatoxin levels in bokina beverage from Ashaiman. The broken horizontal line (---) indicates the acceptable limit of aflatoxin concentration in foods. The three sets of bars on the chart for each of the months represent three different locations where samples were collected.

**Figure 2:**
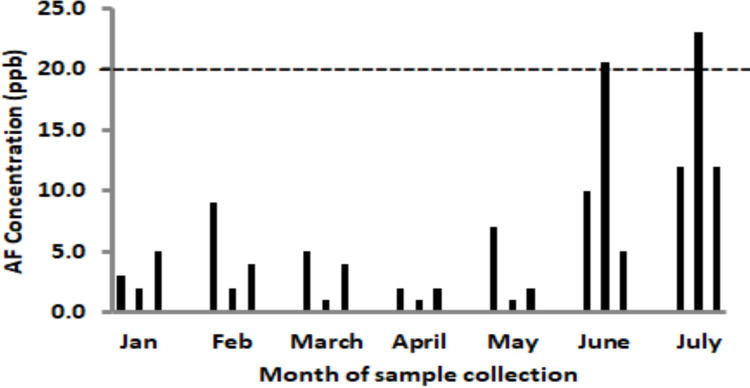
Aflatoxin levels in bokina beverage from Nima. The broken horizontal line (---) indicates the acceptable limit of aflatoxin concentration in foods. The three sets of bars on the chart for each of the months represent three different locations where samples were collected.

Aflatoxin concentrations in the samples varied between sites and collection months (Figures 1 and 2). Low levels of aflatoxins were measured in two months of the dry season, January and February, as well as the first three months of the rainy season, March to May, from both Ashiaman and Nima. Figures 1 and 2 show that generally, the highest levels of aflatoxins in bokina were recorded during the peak period of the rainy season, June and July (2014).^24^

The mean value of total aflatoxin was 12 ppb for samples from Ashaiman and 13.2 ppb for Nima samples. Figure 1 shows that site 1 in Ashaiman recorded the highest level of total aflatoxins (>20 ppb) in June. On the other hand, site 2 in Nima recorded the highest levels in June and July (Figure 2).

From January to May total aflatoxin levels were below 10 ppb for samples from both Ashaiman and Nima (Figures 1 and 2).

## Discussion

Bokina is quite popular in Ghana and is consumed by both adults and children. It can be used as weaning food for children over one-year-old, considering the nutritional value of its components. We investigated aflatoxin levels in bokina, and also determined the levels in millet and milk, from respective producers, which are used to prepare the beverage. In Ghana, millet is mainly obtained from the northern regions and sometimes imported from countries such as Burkina Faso and Mali. However, dairy milk is generally obtained from cattle farmers in Accra and Tema metropolitan areas.

The current study has shown that all bokina samples obtained from commercial producers at both Nima and Ashaiman were contaminated with aflatoxins. The highest levels of aflatoxin contamination were recorded within the rainy season, June and July.

Aflatoxin exposure early in life has been associated with impaired growth, particularly stunting.^25^,^26^ This early exposure is a potential risk for synergistic interactions with other toxins as subjects grow in later years.^27^ Chronic exposure to aflatoxin could also result in liver cancer in individuals infected with the hepatitis B virus.^4^

Aflatoxin M_1_ is the metabolic breakdown product of AFB_1_ and could be present in the milk of lactating cows consuming significant quantities of aflatoxin B_1_ in their diets.^28^,^29^ Seasonal variations of AFM_1_ content of milk have been reported by some investigators.^29^,^30^ To some extent, these seasonal variations can be attributed to cows feeding on less polluted feed during the summer.^30^,^31^

In the present study, aflatoxin was detected in all the samples. Aflatoxin M_1_ levels of milk samples were high, with 57.1%of samples from Ashaiman and 47.6% of samples from Nima (n=21) having levels above the acceptable limit of 0.5 ppb, according to the United States of America Food and Drugs Administration for dairy milk.^12^ This suggests that the milk samples have the potential to cause harm to humans, if they are consumed frequently. Both AFB1 and AFM1 are classified as Group 1 carcinogens, although AFM1 is considered ten times less carcinogenic than AFB1 in animals.^32^ Beside the risk of carcinogenicity and stunting which is a well-known risk marker of poor development of children and proof of chronic malnutrition have been associated with chronic aflatoxin exposure.^25^,^33^ Therefore, dairy milk with an aflatoxin level above the acceptable limit should not be consumed.

Total aflatoxin levels in bokina samples were less than the levels in the millet samples. This was possibly due to the lower level of aflatoxin in the milk compared to the millet which diluted the level in the millet. Thus, our findings suggest that the aflatoxin levels in the bokina samples were due to the high aflatoxin levels in the millet samples. One site in Ashaiman and two sites in Nima reported aflatoxin levels above the limit of 20 ppb. A similar study in Nigeria showed levels of aflatoxin in millet ranging from 34.00 to 40.30 ppb with a mean value of 37.52 ppb.^34^ Millet samples collected during the rainy season were contaminated with AFB_1_ at concentrations between 1,370.28 and 3,495.10 ppb. Although the highest levels of aflatoxin measured in the bokina samples in the present study were around the permissible limit, it is important to note that all doses of aflatoxin have cumulative effect on the risk of cancer.^7^ Aflatoxins have been reported to be responsible for 4.6% to 28.2% of cases of hepatocellular carcinoma globally.^35^ Therefore, the presence of aflatoxins in bokina beverage has the potential to cause toxicity particularly in chronic consumers of the product. Also, since susceptibility to aflatoxin toxicity is highest in the young, children must not be fed with bokina that is contaminated with aflatoxin.^25^

Aflatoxin concentrations in the samples varied between sites and collection months. The southern parts of Ghana have two rainy seasons: the major season is from March to July and a minor season is from September to November, and June records the highest rainfall.^24^

Temperatures in the range of 26.7 to 37.8°C and 18% moisture are optimum for the growth *Aspergillus flavus* to produce aflatoxin.^36^ However, moisture levels in cereals below 12 to 13% inhibit the growth of the fungi at any temperature. In the current study, the highest levels of aflatoxins in bokina and millet were recorded within the rainy season, with peaks in June and July. This was because temperature and humidity levels during rainy seasons were optimum for the growth of *A. flavus*.^36^ Previous studies found reported higher levels of aflatoxin in milk and other foods during the rainy season compared to the dry season.^37^,^38^

It is therefore important to highlight the need for good agricultural handling and storage to minimize the risk of mould growth and mycotoxin contamination of agricultural produce and safeguard public health.^37^ Although the study was conducted over a period of seven months, covering five months of the rainy season and two months of the dry season, the peak periods of both seasons were within the collection period, therefore the effect of the two seasons on aflatoxin level in bokina is represented in our study.

A limitation of the study is that samples were not collected during the remaining five months of the year (of the study), August to December to determine the exposure of bokina and its ingredients to aflatoxins during this period.

Farmers and bokina producers must be periodically educated on good storage methods which they should adopt as preventive and control measures, to reduce exposure the food products to AFB1 and AFM_1_. This will ensure the safety of dairy milk and bokina for human consumption, especially for infants and children. All doses of aflatoxins have progressive effect on the risk of cancer, therefore, monitoring of bokina and similar foods and beverages on the market is important for protection of the public from aflatoxin toxicity.

## Conclusion

The results of this study suggest that the millet used in bokina preparation is exposed to aflatoxin which was transferred from the millet to bokina.
